# Thyroid hormones are associated with pituitary and thyroid volume in a population-based study

**DOI:** 10.1210/jendso/bvag060

**Published:** 2026-03-20

**Authors:** Annemarie Luise Weihs, Katharina Wittfeld, Robin Bülow, Mark Oliver Wielpütz, Matthias Nauck, Henry Völzke, Hans Jörgen Grabe, Alexander Teumer

**Affiliations:** Department of Psychiatry and Psychotherapy, University Medicine Greifswald, Greifswald D-17475, Germany; Department of Psychiatry and Psychotherapy, University Medicine Greifswald, Greifswald D-17475, Germany; Institute of Diagnostic Radiology and Neuroradiology, University Medicine Greifswald, Greifswald D-17475, Germany; Institute of Diagnostic Radiology and Neuroradiology, University Medicine Greifswald, Greifswald D-17475, Germany; Institute of Clinical Chemistry and Laboratory Medicine, University Medicine Greifswald, Greifswald D-17475, Germany; German Centre for Cardiovascular Research (DZHK), Partner Site North, Greifswald D-17475, Germany; German Centre for Cardiovascular Research (DZHK), Partner Site North, Greifswald D-17475, Germany; Institute for Community Medicine, University Medicine Greifswald, Greifswald D-17475, Germany; Department of Psychiatry and Psychotherapy, University Medicine Greifswald, Greifswald D-17475, Germany; German Center for Neurodegenerative Diseases (DZNE), Site Rostock/Greifswald, Greifswald D-17475, Germany; Department of Psychiatry and Psychotherapy, University Medicine Greifswald, Greifswald D-17475, Germany; German Centre for Cardiovascular Research (DZHK), Partner Site North, Greifswald D-17475, Germany

**Keywords:** TSH, T3, T4, hypothalamus, pituitary, thyroid

## Abstract

There is increasing evidence that even a small variation in thyroid function, even within the reference range, is associated with adverse clinical outcomes. Furthermore, there exists a substantial interindividual variation of thyroid function even within euthyroid individuals suggesting an individual hypothalamus-pituitary-thyroid (HPT) axis setpoint. Here we assess potential contributions of the HPT axis organ volumes to the variation of thyroid hormones in the general population.

Using linear regression models, we analyzed the association of circulating free T3, free T4, and log-transformed TSH levels with hypothalamus, pituitary gland, and log-transformed thyroid volume in a subsample of up to n = 3438 participants from the population-based Study of Health in Pomerania with a mean age 50.6 years, 44.6% female, and a mean TSH 1.38mU/L.

Performing cross-sectional analyses at baseline, we observed inverse associations of pituitary volume with free T4 levels (β=−0.74,P=0.017,n=1,372) and of thyroid volume with TSH levels ($\beta =-0.72,\;P=3.1\times 10^{-147},\;n=3,430$), as well as positive associations of thyroid volume with free T3 ($\beta =0.11,\;P=1.3\times 10^{-4},\;n=3,310$) and free T4 levels ($\beta =0.81,\;P=1.2\times 10^{-18},\;n=3,311$) but observed no association regarding hypothalamus volume (n ≤ 1636). The findings for free T3 and free T4 were supported by longitudinal analyses in a subsample of up to n = 2040 participants with available longitudinal data and a mean follow-up time of 7.3 years.

In summary, using data from a population-based sample, we identified associations between pituitary volume and circulating free T4 as well as thyroid gland volume with circulating levels of TSH, free T3, and free T4 contributing to the variation of thyroid hormone levels.

Thyroid hormones are essential regulatory molecules with critical roles in growth, development, and metabolism [1, 2]. They control, among others, weight gain, cell differentiation, and development of the central nervous system, lungs, and heart in the fetus and infant, and oversee, for example, cholesterol metabolism, bone formation, and brain organization and function throughout life [1, 2]. Their vital role for the metabolism of almost all body tissues and their pleiotropic effects make them essential for survival and normal functioning of the human body [1].

The synthesis and secretion of thyroid hormones is tightly regulated by the hypothalamus-pituitary-thyroid (HPT) axis through a negative feedback loop [1, 2]. TRH is synthesized in the hypothalamus and transported to the pituitary where it promotes the release and new production of TSH [1]. TSH in turn stimulates the thyroid to synthesize and release T4 and to a lesser extent T3 into the circulation, whose increased concentrations suppress TRH production [1]. When assessing thyroid function, the circulating hormone levels are measured with increased TSH and decreased free T3 (fT3) and/or free T4 (fT4) levels indicating hypothyroidism, while the inverse combination indicates hyperthyroidism.

In the past decade, it has become clear that not only overt and subclinical hypo- and hyperthyroidism, affecting approximately 10% of individuals over their lifespan [3], but even small differences in thyroid function, even within the reference range, are associated with clinical consequences such as atrial fibrillation, coronary heart disease, stroke, and depression as well as cardiovascular and overall mortality [4-9]. Furthermore, there exists a substantial interindividual variation of thyroid function even within healthy individuals suggesting an individual HPT axis setpoint [10]. Despite the physiological significance of thyroid hormones as well as the clinical importance and high prevalence of thyroid dysfunction, many factors contributing to the interindividual variation of thyroid hormones are still unknown. A few studies on the association of thyroid function with subcortical and cortical brain structures in the general population have been published, but little is known about the variation of hormone levels specifically related to the HPT axis organs. In detail, associations of measured TSH and fT4 blood levels and genetically determined hypothyroidism status with intracranial, total brain, white matter, hippocampus, cerebellar, and pallidum grey matter volume have been reported [11-13].

Regarding the involvement of the HPT axis, case reports have repeatedly pointed to pituitary hyperplasia secondary to primary hypothyroidism in both adults [14] and children [15], with the hyperplasia often resolving after thyroid hormone therapy. These reports, however, showcase extreme examples with limited generalizability to the population-based variability of thyroid function, ie, at euthyroid and subclinical levels. Concerning thyroid gland volume, a few previous studies have pointed to an inverse association with TSH levels [16-21]; however, most of those studies are case-control comparisons, focus only on TSH, and control sparsely for potential confounders. Analyses on hypothalamus volume are missing, but 1 study analyzed its association with TSH, fT3, and fT4 in a small sample of hyperthyroidism patients [22]. In addition, due to the diverse methodology and partial scarcity of the literature, estimations on the magnitude of the effects, which might be relevant for refining the individual HPT axis setpoint, are lacking.

In the present study, we tested the association of TSH, fT3, and fT4 with the volumes of the hypothalamus, pituitary, and thyroid gland in a large population-based sample from a region in northeast Germany to assess whether effects seen in patients are similarly observable in the general population and to fill research gaps regarding variation of fT3 and fT4 levels while giving first estimates on the effect magnitudes.

## Materials and methods

### Cohort description

The Study of Health in Pomerania (SHIP) is a population-based cohort study in Western Pomerania, a region in northeast Germany, assessing the prevalence and incidence of common population-relevant diseases and their risk factors [23]. For the current analysis, data from the SHIP-TREND cohort was used. The dataset from the baseline examination (SHIP-TREND-0, assessed between 2008 and 2012; ages 20-84 years) comprises 4420 individuals randomly drawn from the adult population of Western Pomerania, Germany [23]. The dataset from the first follow-up (SHIP-TREND-1, assessed between 2016 and 2019) comprises 2507 individuals. All participants gave written informed consent. The study conformed to the principles of the Declaration of Helsinki, and the medical ethics committee of the University of Greifswald approved the study protocol.

### Measuring TSH, fT3, and fT4

In SHIP-TREND, the thyroid-related hormones (TSH: mU/L, fT3: pmol/L, fT4: pmol/L) were assessed out of serum for all participants using electrochemiluminescence immunoassays (Dimension Vista, Siemens Healthcare Diagnostic, Erlangen, Germany; TSH: Siemens Cat# K6412, RRID:AB_3740175; fT3: Siemens Cat# K6416, RRID:AB_2924986; fT4: Siemens Cat# K6410, RRID:AB_2801666). Serum aliquots were prepared for immediate analysis and for storage at −80 °C in the Integrated Research Biobank (Liconic, Liechtenstein). The reference range of TSH in SHIP-TREND-0 has previously [24] been established as [0.49; 3.29] mU/L. Serum autoantibodies to thyroperoxidase were measured by an enzyme immunoassay (VARELISA, Elias Medizintechnik GmbH, Freiburg, Germany; Roche Cat# 06368590190, RRID:AB_2916057) using the cutoffs as described before [25].

### Measuring thyroid gland volume

In SHIP-TREND, ultrasonography was performed on the same day as the blood draw with a portable device using a 13-MHz linear array transducer (Vivid I, General Electric, Chicago, IL, USA). Intra- and interobserver reliabilities were assessed before the start of the study and semiannually during the study. For thyroid volume, all interobserver and interdevice variabilities showed mean differences (±2 SD) of < 5% (<25%) [26]. Thyroid volume (cm^3^) was calculated as length × width × depth × 0.479 for each lobe [27].

### Measuring hypothalamus, pituitary, and total intracranial volume

In SHIP-TREND-0 and SHIP-TREND-1, whole-body magnetic resonance imaging (MRI) has been offered to all, except upon contraindication, and brain scans were performed on n = 2154 (SHIP-TREND-0) and n = 1421 (SHIP-TREND-1) participants using a 1.5-Tesla scanner (Magnetom Avanto, Siemens Healthineers, Erlangen, Germany) [28]. Standardized examinations were executed by 2 trained technicians.

For the T1-weighted magnetization prepared rapid acquisition gradient-echo sequence, the following parameters were used: axial plane, repetition time = 1900ms, echo time = 3.4 ms, flip angle = 15°, and resolution = 1.0 × 1.0 × 1.0 mm [3] [28]. For more details, see [28]. The hypothalamus (cm^3^) and total intracranial volumes of SHIP-TREND were extracted using the fully automated recon-all pipeline of FreeSurfer 7.3.2 [29].

The pituitary volume (cm^3^) of both SHIP-TREND datasets was manually extracted via slice-by-slice segmentation performed in the coronal orientation of magnetization prepared rapid acquisition gradient-echo using OsiriX DICOM Viewer v.3.7.1 32-bit (Pixmeo SARL, Bernex, Switzerland) [30] using the method and data described before [31]. Briefly, the observer verified the ventral and dorsal boundaries of the pituitary gland using a sagittal T2-weighted turbo-spin echo sequence of the head with a voxel size of 1.2 × 0.9 × 3.0 mm^3^ and the following imaging parameters: repetition time = 2610 ms, echo time = 102 ms, field of view = 240 × 240 mm^2^, matrix = 256 × 192, slice gap = 0.45 mm, flip angle = 180°, and bandwidth = 190 Hz/pixel. The pituitary volume was determined by 1 observer after training by a radiologist with 8 years of imaging experience. Imaging quality and other remarks were recorded for quality assessment.

### Statistical analyses

All statistical analyses were performed using R version 4.3 [32].

In the first analysis step, we used linear regression models to check for associations between the volumetric measures (exposures) and hormone levels (outcomes) in SHIP-TREND-0 (baseline) adjusting for age at blood draw (years), sex, age × sex, body mass index (kg/m^2^), current smoking status (yes/no), self-reported average daily alcohol intake within the last 30 days (ethanol in g/d), years of education (years), fasting status (≤8 hours, >8 hours), time of blood draw, weekday of blood draw and study week, and additionally total intracranial volume (dm^3^) in the hypothalamus and pituitary analyses and thyroperoxidase antibody positivity in the thyroid gland volume analyses.

Where noted, sensitivity analyses additionally included serum total/high-density lipoprotein cholesterol ratio, diastolic and systolic blood pressure (mmHg), and diabetes status (yes/no). To account for nonlinear dependencies, age was modeled using restricted cubic splines with 3 knots in all analyses. The number of education years was derived from the highest school and professional degrees with those participants without a finished degree set to 8 years, the minimal number among the remaining participants. To reduce the skewness of the distribution, the TSH levels and thyroid gland volumes were natural log-transformed in the cross-sectional analyses (including for calculation of the explained variance). For the other measurements, the untransformed values were included in the association models. As hypothalamus, pituitary, and thyroid volume were considered independent exposures, analyses with Bonferroni-corrected *P* < .05/3 = .017 were considered statistically significant.

Significant results underwent a second step to assess their effects over time. Here, we used linear regression models to check if time-normalized volume differences between baseline and first follow-up (exposure) are associated with time-normalized hormone level differences (outcome). Time normalization was performed by dividing the difference of the respective measurements between baseline and follow-up (ie, SHIP-TREND-1 – SHIP-TREND-0) by the number of years between both time points. The models were adjusted for baseline age at blood draw (nonlinearly), sex and age × sex, the pituitary analysis additionally for baseline intracranial volume, and the thyroid analysis additionally for thyroperoxidase antibody positivity. In this second step, associations with *P* < .05 were considered statistically significant.

## Results

### Study population

**Figure 1:**
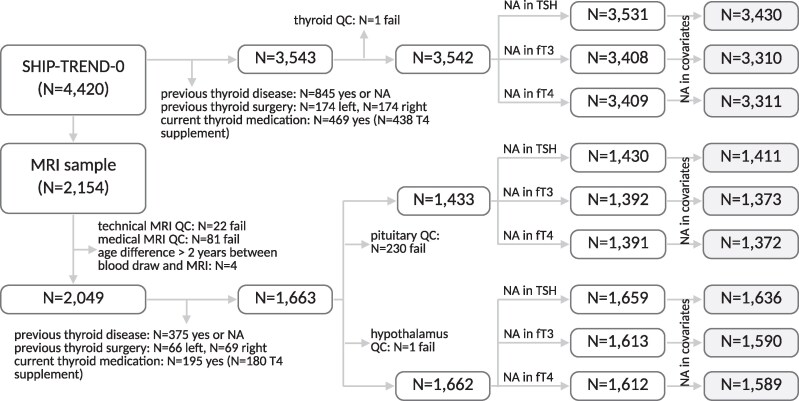
Overview of the inclusion and exclusion criteria. For the main analyses of step 1 (marked in grey), we have used data from n = 3438 participants of SHIP-TREND-0. The figure was created in BioRender. Kuehn, L. (2026) https://BioRender.com/aco2gb7. Abbreviations: fT3, free T3; fT4, free T4; MRI, magnetic resonance imaging; NA, not available; QC, quality control.

Figure 1 provides an overview of the inclusion and exclusion criteria in the current study. In the hypothalamus and pituitary analyses, individuals with poor quality MRI scans, individuals with medical conditions such as a history of cerebral tumor, stroke, Parkinson's disease, multiple sclerosis, epilepsy, hydrocephalus, enlarged ventricles, or pathologic lesions, as well as individuals with more than 2 years between blood sampling and MRI were excluded. The resulting sample had a median time difference of 17 days between blood draw and MRI. In the pituitary analyses, individuals with remarks on difficulties during the pituitary volume extraction were additionally excluded. Unless stated otherwise, participants with previous thyroid disease or operation or who were taking thyroid medication at baseline (Anatomical Therapeutic Chemical code H03, mostly levothyroxine treatment) were excluded from all analyses.

**Figure 2:**
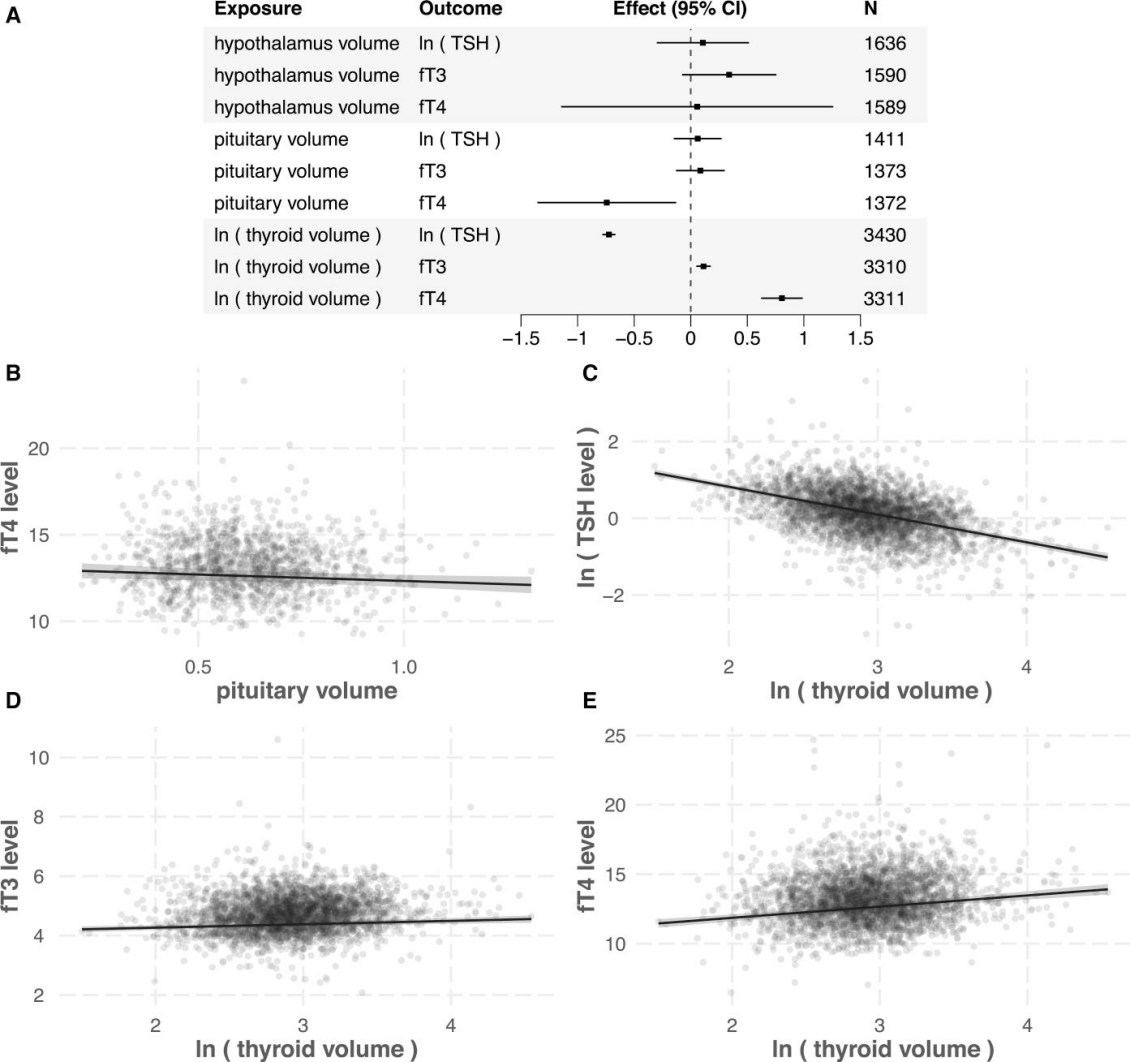
(A) Forest plot of the main analyses of step 1. (B-E) Effect plots of the significant models in step 1 (cross-sectional analyses). All continuous variables except the plotted predictor are set to their mean value, all factors are set to base level, and all logical variables are set to false. (B) Pituitary volume was inversely associated with the log-transformed free T4 levels (β=−0.74[−1.35;−0.13], n=1,372). Log-transformed thyroid volume was inversely associated with (C) log-transformed TSH levels (β=−0.72[−0.78;−0.67], n=3,430) and positively associated with (D) free T3 levels (β=0.11[0.055;0.17], n=3,310) and (E) free T4 levels (β=0.81[0.63;0.98], n=3,311).

For the main analyses of step 1, data from n = 3438 participants of the SHIP-TREND-0 sample remained after quality control. The variable distributions of the samples used for the pituitary and hypothalamus main analyses and the thyroid gland main analyses are shown in Table 1. The resulting plots of the significant models as well as a graphical summary of the results are provided in Fig. 2. Data of n = 2251 participants were used in the longitudinal analysis. Their mean time difference between baseline and follow-up was 7.3 years ± 0.66.

**Table 1:** Sample description of the study populations

Variable	Pituitary/hypothalamus main analyses step 1 (n = 1637)		Thyroid gland main analyses step 1 (n = 3433)	
	Mean (SD)/n (%)	#NA	Mean (SD)/n (%)	#NA
TSH (mU/L), mean (SD)	1.34 (0.85)	1	1.38 (1.13)	3
fT3 (pmol/L), mean (SD)	4.77 (0.59)	47	4.73 (0.61)	123
fT4 (pmol/L), mean (SD)	13.18 (1.58)	48	13.20 (1.71)	122
Pituitary volume (cm^3^), mean (SD)	0.61 (0.15)	222	—	—
Hypothalamus volume (cm^3^), mean (SD)	0.77 (0.08)	0	—	—
Thyroid volume (cm^3^), mean (SD)	—	—	19.87 (8.06)	0
Age at blood draw (years), mean (SD)	49.7 (14.1)	0	50.6 (15.5)	0
Sex (female), n (%)	732 (44.7)	0	1532 (44.6)	0
BMI (kg/m^2^), mean (SD)	27.50 (4.39)	0	27.94 (5.08)	0
Smoking status (yes), n (%)	406 (24.8)	0	966 (28.1)	0
Alcohol intake (ethanol in g/d), mean (SD)	8.80 (12.63)	0	9.29 (14.07)	0
Education years, mean (SD)	12.75 (2.36)	0	12.32 (2.36)	0
Total intracranial volume (dm^3^), mean (SD)	1.60 (0.16)	0	—	—
Fasting status (>8 hours), n (%)	1198 (73.2)	0	2124 (61.9)	0
Hour of blood draw, mean (SD)	8.70 (0.96)	0	8.76 (1.08)	0
TPO antibody positivity (yes), n (%)	95 (5.8)	5	188 (5.5)	0

Step 1 refers to the cross-sectional analyses. Numerical variables are listed as mean (SD), categorical variables as number (percentage). TPO antibody positivity was defined as >60 kIU/L for men and >100 kIU/L for women as established in [25] for this cohort.

Abbreviations: #NA, number of missing values; BMI, body mass index; fT3, free T3; fT4, free T4; TPO, thyroperoxidase.

### Hypothalamus volume

As depicted in Table 2, there was no statistically significant association of hypothalamus volume with any of the 3 hormone levels.

**Table 2:** Association results of hypothalamus volume with TSH, fT3, and fT4 levels

	Analysis description	Outcome	Effect [95% CI]	*P*-value	n
1	Cross-sectional base model	ln(TSH)	0.107 [−0.295; 0.509]	0.60	1636
2	Cross-sectional base model	fT3	0.340 [−0.071; 0.751]	0.11	1590
3	Cross-sectional base model	fT4	0.057 [−1.140; 1.254]	0.93	1589

The association results of the cross-sectional analyses of the hormone levels and the hypothalamus volume in SHIP-TREND-0 (baseline) adjusting for age at blood draw, sex, age × sex, body mass index, current smoking status, self-reported average daily alcohol intake within the last 30 days, years of education, fasting status, time of blood draw, weekday of blood draw, study week, and total intracranial volume are presented.

Abbreviations: CI, confidence interval; fT3, free T3; fT4, free T4.

### Pituitary volume

As illustrated in Table 3, pituitary volume was inversely associated with fT4 levels with a *P*-value that was at the predefined significance level (β=−0.74[−1.4;−0.13], P=0.017, n=1,372, analysis 3). This effect estimate was robust in the following sensitivity analyses. Of the n=1,372 participants, n=90 had TSH levels below and n=33 above the reference range [0.49;3.29] (n=1 unknown). When restricting the sample to those individuals with TSH within the reference range (analysis 4), the effect size remained similar. Restricting the sample to those individuals with a maximum time difference of 7 days (analysis 5) or 0 days (analysis 6) between blood draw and MRI yielded the same effect direction, with the effect size varying due to the substantial reduction of participants. Adjusting the model for the cardiovascular risk factors serum total/high-density lipoprotein cholesterol ratio, diastolic and systolic blood pressure, and diabetes status (analysis 7) also did not notably change the effect size. The same holds for adjusting the base model for TSH levels (analysis 8), indicating that TSH does not substantially mediate the association. All sensitivity analyses except analysis 6 yielded nominally statistically significant effect sizes. The pituitary volume explained 0.38% of the variance in fT4 levels.

**Table 3:** Association results of pituitary volume with TSH, fT3, and fT4 levels

Analysis description		Outcome	Effect [95% CI]	*P*-value	n
Main analyses					
1	Cross-sectional base model	ln(TSH)	0.062 [−0.145; 0.268]	0.56	1411
2	Cross-sectional base model	fT3	0.085 [−0.125; 0.295]	0.43	1373
3	Cross-sectional base model	fT4	−0.742 [−1.350; −0.134]	0.017	1372
Sensitivity analyses					
4	Only participants with TSH in reference range	fT4	−0.678 [−1.314; −0.042]	0.037	1248
5	Time difference ≤ 7 days	fT4	−1.433 [−2.382; −0.483]	0.003	501
6	Time difference = 0 days	fT4	−1.264 [−2.792; 0.263]	0.104	221
7	Additional adjustment for cardiovascular risk factors	fT4	−0.704 [−1.318; −0.090]	0.025	1369
8	Additional adjustment for TSH levels	fT4	−0.718 [−1.321; −0.114]	0.020	1371

The association results of the cross-sectional analyses of the hormone levels and the pituitary volume in SHIP-TREND-0 (baseline) adjusting for age at blood draw, sex, age × sex, body mass index, current smoking status, self-reported average daily alcohol intake within the last 30 days, years of education, fasting status, time of blood draw, weekday of blood draw, study week, and total intracranial volume are presented. Additional analyses were conducted excluding participants with TSH outside the reference range (4), excluding participants with more than 7 days (5) and more than 0 days (6) between blood draw and magnetic resonance imaging and when additionally adjusting for cardiovascular risk factors (7) and TSH levels (8).

Abbreviations: CI, confidence interval; fT3, free T3; fT4, free T4.

To further validate the association between pituitary volume and fT4 levels, we checked if the pituitary volume difference between baseline (SHIP-TREND-0) and follow-up (SHIP-TREND-1) is associated with the fT4 level difference (Methods, step 2). Using the n = 934 participants who had quality-controlled pituitary volume data and fT4 levels available in both baseline and follow-up, we found an inverse association between pituitary volume difference and fT4 level difference (β=−1.42[−2.49;−0.35], P=0.009, n=934). The effect direction was unchanged when excluding those participants who had previous thyroid surgery or were taking thyroid medication at baseline or follow-up (β=−1.18[−2.27;−0.10], P=0.032, n=785). All remaining participants had declared previous thyroid disease. Note that this longitudinal analysis showed the same effect direction as the initial cross-sectional at baseline.

There was no significant association of pituitary volume with TSH or fT3 levels (Table 3, analyses 1 and 2).

### Thyroid volume

As shown in Table 4, there was a strong inverse association between thyroid volume and TSH levels at baseline (β=−0.72[−0.78;−0.67], $P=3.1\times 10^{-147}$, n=3,430, analysis 1). Accordingly, a 10% increase in thyroid volume corresponds to a $1-(1.1{)}^{-0.72}=6.6\text{\%}$ decrease in TSH levels. Additionally, we identified significant positive associations of thyroid volume with fT3 levels (β=0.11[0.055;0.17], $P=1.3\times 10^{-4}$, n=3,310, analysis 2) and fT4 levels (β=0.81[0.63;0.98], $P=1.2\times 10^{-18}$, n=3,311, analysis 3). Accordingly, a 10% increase in thyroid volume corresponds to a 0.11×log1.10=0.01 pmol/L increase in fT3 levels and a 0.81×log1.10=0.08 pmol/L increase in fT4 levels, respectively. The effect sizes did not notably change when excluding participants whose TSH levels were above or below the reference range (analyses 4, 7, 10), when expanding the sample to those participants taking thyroid medication at baseline (analyses 5, 8, 11), or when adjusting the models for additional cardiovascular risk factors (analyses 6, 9, 12). Thyroid volume explained 15.53%, 0.35%, and 2.22% of the variance in TSH, fT3, and fT4 levels, respectively. For comparison, TSH levels explained 0.20% and 1.33% of the variance in fT3 and fT4 levels, respectively.

**Table 4:** Association results of thyroid volume with TSH, fT3, and fT4 levels

Analysis description		Outcome	Effect [95% CI]	*P*-value	n
Main analyses					
1	Cross-sectional base model	ln(TSH)	−0.723 [−0.775; −0.671]	3.1E-147	3430
2	Cross-sectional base model	fT3	0.113 [0.055; 0.172]	1.3E-4	3310
3	Cross-sectional base model	fT4	0.806 [0.628; 0.984]	1.2E-18	3311
Sensitivity analyses					
4	Only participants with TSH in reference range	ln(TSH)	−0.505 [−0.551; −0.460]	2.3E-98	3113
5	Including participants on thyroid medication	ln(TSH)	−0.724 [−0.776; −0.671]	7.4E-147	3455
6	Additional adjustment for cardiovascular risk factors	ln(TSH)	−0.725 [−0.777; −0.673]	2.0E-147	3423
7	Only participants with TSH in reference range	fT3	0.072 [0.009; 0.134]	0.025	3002
8	Including participants on thyroid medication	fT3	0.114 [0.056; 0.172]	1.2E-4	3333
9	Additional adjustment for cardiovascular risk factors	fT3	0.113 [0.055; 0.171]	1.3E-4	3304
10	Only participants with TSH in reference range	fT4	0.753 [0.558; 0.948]	4.7E-14	3003
11	Including participants on thyroid medication	fT4	0.790 [0.613; 0.967]	3.7E-18	3334
12	Additional adjustment for cardiovascular risk factors	fT4	0.803 [0.625; 0.982]	1.7E-18	3305

The association results of the cross-sectional analyses of the hormone levels and the thyroid gland volume in SHIP-TREND-0 (baseline) adjusting for age at blood draw, sex, age × sex, body mass index, current smoking status, self-reported average daily alcohol intake within the last 30 days, years of education, fasting status, time of blood draw, weekday of blood draw, study week, and thyroperoxidase antibody positivity are presented. Additional analyses were conducted excluding participants with TSH outside the reference range (4, 7, 10), including participants currently taking thyroid medication (5, 8, 11), and when additionally adjusting for cardiovascular risk factors (6, 9, 12).

Abbreviations: CI, confidence interval; fT3, free T3; fT4, free T4.

In step 2 (see Methods), we checked if the thyroid volume difference between SHIP-TREND-0 and SHIP-TREND-1 is associated with the hormone level differences. Using all participants with quality-controlled thyroid volume and hormone levels available in both baseline and follow-up, who had no previous thyroid surgery and were not taking thyroid medication at baseline or follow-up, we found no significant association of thyroid volume difference with TSH level difference (β=−0.0026[−0.0090;0.0038], P=0.43, n=2,096). On the other hand, we found positive associations with fT3 level difference (β=0.0097[0.0025;0.017], P=0.0082, n=2,040) and fT4 level difference (β=0.020[0.0038;0.036], P=0.016, n=2,036). All those participants had declared previous thyroid disease. Again, note that for fT3 and fT4, step 2 showed the same effect direction as the initial cross-sectional analysis.

## Discussion

Despite the physiological significance of thyroid hormones, as well as the prevalence and clinical importance of thyroid dysfunction, many key players in the regulation of thyroid hormone levels still need to be elucidated. In the present study, we have analyzed the associations between volumes and hormone levels of the HPT axis. In the first step, using data from SHIP-TREND-0 (baseline), we identified inverse associations of pituitary volume with fT4 levels and of thyroid volume with TSH levels, as well as positive associations of thyroid volume with fT3 and fT4 levels. On the other hand, our data showed no association between hypothalamus volume and TSH, fT3, or fT4 levels or between pituitary volume and TSH or fT3 levels. In the second step, we could validate the associations of pituitary volume with fT4 level and of thyroid volume with fT3 and fT4 but not TSH levels by considering the time-normalized volume and hormone level differences between SHIP-TREND-0 and SHIP-TREND-1 (follow-up). The study data is from an area of mild iodine deficiency [33].

### Hypothalamus

Information on the association between hypothalamus volume and levels of TSH, fT3, and fT4 is scarce. A 2024 study comparing 19 newly diagnosed untreated hyperthyroid patients (high fT3 and fT4 and reduced TSH levels) with 15 age- and sex-matched controls found no significant difference in hypothalamus volume between the 2 groups [22]. The volume was also not associated with the hormone levels directly, neither in the whole nor in the patient or control subsamples [22]. To the best of our knowledge, this is the only study analyzing this relationship, and despite a substantially larger sample size, we also found no association between hypothalamus volume and hormone levels. There are several possibilities for this lack of significant associations. It could be perceivable that the hormone levels are not associated with the volume of the whole hypothalamus but rather the proportions of the hypothalamus subunits, which could not be assessed in our study due to limited MRI resolution. Relatedly, the hormone levels might be associated with cell composition of the hypothalamus rather than its volume. And, of course, it is possible that an association was not observed because our sample size was still not large enough or that there really is no association between hypothalamus volume and the hormones.

### Pituitary

Changes in pituitary volume are not uncommon and are suspected to usually be attributed to physiological reactions to external stimuli [34]. For example, accelerated growth is observed in girls during puberty [35], shrinkage occurs with age [31], and hyperplasia is normal during pregnancy, reversing itself afterwards [34]. Also, pituitary hyperplasia secondary to primary hypothyroidism has frequently been mentioned in case reports, both in adults [14] and children [15], with the hyperplasia often resolving after thyroid hormone therapy, thus avoiding neurosurgery for some patients. While these reports display extreme cases, studies within the general population are lacking. One reason is that there are currently no well-established tools for automated pituitary volume extraction in large MRI datasets. Our study addresses this gap by illustrating that comparable associations between thyroid hormone levels (here fT4) and pituitary volume can already be observed in the general population, ie, at a subclinical level. As we had only n = 4 individuals taking levothyroxine medication, having pituitary volume measurements, and passing all inclusion criteria, we could not test the possible effects of T4 treatment on our observed associations. Furthermore, the nonsignificant association of TSH with the volume of the TSH-producing pituitary gland could indicate that the effect of fT4 with the pituitary is not a direct result of the variation in TSH levels. Consequently, the pituitary volume could act as a contributor to the HPT axis setpoint, keeping TSH at an individual-specific level. However, larger datasets and methods assessing causality are necessary to get deeper insights into this relationship in the general population.

### Thyroid

Regarding the thyroid gland, some previous studies have pointed to relations between thyroid volume and levels of TSH, fT3, and fT4. For example, a common cause of hyperthyroidism is Graves's disease, characterized by an enlarged thyroid gland and excessive thyroid hormone secretion [36]. When analyzing patients with untreated Hashimoto thyroiditis, Kawasaki et al [37] found higher serum fT3 levels in patients with moderate to large goiter compared to euthyroid controls. Also, positive correlations of thyroid volume or goiter presence with fT4 levels [16], as well as inverse correlations with TSH levels [16-21] have been observed. In a 2008 study, patients with incident overt autoimmune hypothyroidism (elevated serum TSH and reduced serum total T4 levels) had lower median thyroid volumes compared to controls [17], which is in line with our findings. And in a comparison of adults whose TSH levels rose above a threshold within 5 years of the baseline examination and those whose TSH levels stayed below, smaller thyroid volume at baseline was associated with future TSH elevation [38]. Most of those studies, however, are case-control comparisons [17, 18, 37, 38] or focus only on TSH [16, 19-21, 38]. Also, covariates are often not included in the models [16, 21, 37, 39, 40] or their incorporation neutralizes the effect [19]. Other studies show no [18, 39, 40] or reversed effect directions. For example, lower fT4 levels were observed in all patients with untreated Hashimoto thyroiditis and elevated TSH levels in those with no to moderate but not large goiter when compared to euthyroid controls [37]. Also, TSH levels were positively associated with thyroid volume in a study of glucose metabolism disorders [41]. The present study strengthens the hypothesis that thyroid volume is inversely associated with TSH and in particular is positively associated with fT3 and fT4 levels. These associations are robust toward the inclusion of covariates and are observable in the general population.

### Limitations

When interpreting these results, at least 3 study limitations have to be kept in mind. First, due to the cross-sectional nature of the analyses, no statement about cause and consequence in the associations can be made. Establishing larger studies combining thyroid hormone measurements and HPT axis-related organ volumes with genome-wide data could provide a future step toward assessing causality, eg, via Mendelian randomization [42]. Second, brain volumes, especially of the pituitary, are dynamic in a short time interval. For that reason, we have excluded participants with a time difference of more than 2 years between blood draw and MRI measurement and ran additional sensitivity analyses on participants with a maximum time difference of 7 days and 0 days, supporting the main analyses. Furthermore, the measures obtained from brain MRI segmentation are prone to measurement errors because of limited resolution using the 1.5Tesla scanner and due to manual segmentation of the pituitary resulting in putatively underestimating the association effect sizes. Future studies using well-trained automatic image segmentation from higher resolution MRI images in larger cohorts are needed to address this limitation. Third, iodine status has an influence on thyroid function in general but could not be taken into account in the current analyses because the measured urinary iodine status in the SHIP cohorts is highly volatile as it is dependent on the participant's specific food intake shortly before blood draw. The hormone levels and imaging measures are more stable. It should be noted that the current study was performed in an area of mild iodine deficiency [33].

## Conclusion

In summary, using data from the SHIP-TREND sample, we identified associations between pituitary volume and circulating fT4 levels as well as thyroid gland volume with circulating levels of TSH, fT3, and fT4. Supporting previous research, which considered mostly a few manifest thyroid disease cases, these associations can even be observed in individuals at a euthyroid to subclinical thyroid disease level. Our observed associations may act as contributors to the individual HPT setpoint definition and to an increased risk for adverse clinical outcomes related to already small changes in thyroid function that were observed among euthyroid individuals.

## Data Availability

Restrictions apply to the availability of some or all data generated or analyzed during this study to preserve patient confidentiality or because they were used under license. The corresponding author will on request detail the restrictions and any conditions under which access to some data may be provided.

## Abbreviations

fT3: free T3
fT4: free T4
HPT: hypothalamus pituitary thyroid
MRI: magnetic resonance imaging
SHIP: Study of Health in Pomerania
